# Hepatitis B virus (HBV) X gene mutations and their association with liver disease progression in HBV-infected patients

**DOI:** 10.18632/oncotarget.22428

**Published:** 2017-11-06

**Authors:** Ahmed A. Al-Qahtani, Mashael R. Al-Anazi, Nyla Nazir, Rohit Ghai, Ayman A. Abdo, Faisal M. Sanai, Waleed K. Al-Hamoudi, Khalid A. Alswat, Hamad I. Al-Ashgar, Mohammed Q. Khan, Ali Albenmousa, Damian Dela Cruz, Marie Fe F. Bohol, Mohammed N. Al-Ahdal

**Affiliations:** ^1^ Department of Infection and Immunity, Research Center, King Faisal Specialist Hospital & Research Center, Riyadh, Saudi Arabia; ^2^ Department of Microbiology and Immunology, Alfaisal University School of Medicine, Riyadh, Saudi Arabia; ^3^ Institute of Hydrobiology, Department of Aquatic Microbial Ecology, Biology Centre of the Academy of Sciences of the Czech Republic, České Budějovice, Czech Republic; ^4^ Section of Gastroenterology, Department of Medicine, College of Medicine, King Saud University, Riyadh, Saudi Arabia; ^5^ Gastroenterology Unit, Department of Medicine, King Abdulaziz Medical City, Jeddah, Saudi Arabia; ^6^ Gastroenterology Unit, Department of Medicine, King Faisal Specialist Hospital & Research Center, Riyadh, Saudi Arabia; ^7^ Department of Gastroenterology, Prince Sultan Medical Military City, Riyadh, Saudi Arabia; ^8^ Liver Disease Research Center, King Saud University, Riyadh, Saudi Arabia

**Keywords:** HCC, hepatitis, cirrhosis, HBx, mutations

## Abstract

Hepatitis B virus (HBV) is one of the most widespread human pathogens causing chronic hepatitis, liver cirrhosis, and hepatocellular carcinoma (HCC). This study investigated the clinical impact of single and combinational mutations in HBx gene on the pathogenesis of HCC during progressive stages of liver disease. The patients were categorized into inactive HBV carriers, active carriers, cirrhosis and HCC groups based on disease severity. Male sex, age > 50 years, and high serum alanine aminotransferase level were associated with risk of progressive liver disease. I127T, V131I, and F132Y/I/R mutations showed a significant increasing trend associated with the disease progression to HCC. H94Y and K130M mutations were also significantly associated with severe liver disease. One double mutation (K130M+V131I) and two triple mutations (I127T+K130M+V131L and K130M+V131I+F132Y) were observed, with significant rising prevalence through progressive clinical phases of liver disease to HCC. Several single and combinational mutations in HBx correlating with severity and progressive clinical phases of HBV infection were identified. The mutational combinations may have a synergistic effect in accelerating the progression to HCC. These specific patterns of HBx mutations can be useful in predicting the clinical outcome of HBV-infected patients and may serve as early markers of high risk of developing HCC.

## INTRODUCTION

Hepatitis B virus (HBV) is the major causative agent of hepatitis worldwide. According to World Health Organization (WHO), an estimated 240 million people globally have chronic HBV infection (http://www.who.int/mediacentre/factsheets/fs204/en/). HBV infection has been reported to result in liver cirrhosis, hepatic failure, or hepatocellular carcinoma (HCC), leading to about 780,000 deaths each year globally [1]. HCC is the fifth most common cancer and the second cause of cancer-related deaths in the world [2], and nearly 53% of HCC is estimated to be related to HBV [3]. Additionally, it is predicted that the risk of HCC increases by 5- to 15-fold in individuals who are chronically infected with HBV [4].

HBV is a small virus with a genome consisting of a circular partially double-stranded DNA of about 3.2 kb in length. The genome is organized into four overlapping open reading frames that encode the viral core protein (capsid), surface proteins (envelope), reverse transcriptase, and non-structural X protein (HBx) [5]. Compared to the other three HBV proteins, a limited number of studies have examined the role of HBx mutations in the oncogenic potential of the virus. HBx protein (154 amino acids) has functionally distinct domains with a strong negative regulatory N-terminus and a transactivation region mapped to its C-terminus [6]. HBx has the ability to activate and/or deactivate both viral and cellular promoters through a direct interaction with nuclear transcription factors or via cytoplasmic signal transduction [7]. HBx is a pleiotropic regulatory molecule exerting its effects on a wide range of cell processes, including cell cycle, cell proliferation, and apoptosis [8]. Several studies have reported the hepatocarcinogenic effect of HBx, while others have suggested that HBx has limited influence on oncogenesis and/or oncogenic signals [7–9]. Interestingly, previous studies have shown that the majority of HBx detected in HCC tissues had mutations that may alter the function of HBx [10, 11]. HBx mutants harboring both point mutations and deletions, especially C-terminal truncations, have been frequently detected in HCC patients [10, 12, 13]. Taken together, these studies suggest that HBx plays a crucial role in the pathogenesis and progression of HBV-related complications.

Several studies have reported the association between HBx mutations and liver cirrhosis and HCC in HBV-infected patients of different ethnicities. Malik et al. (2012) identified mutations at positions 127, 130, and 131 in HBx protein and suggested that they might increase the risk of HCC development [14]. Another study in the Indian population has reported a significantly higher prevalence of mutations at amino acid 88 and a double mutation at positions 130+131 of HBx protein in patients with liver cirrhosis compared to inactive carriers [15]. Similarly, a study in the Chinese population has found that the mutation at amino acid 88 of HBx was highly frequent in HCC tumor samples compared to non-tumor samples [16]. Shinkai et al. (2007) have analyzed the HBx mutations in Japanese patients and concluded that the mutations at amino acid 94, 127, 130, and 131 were more prevalent in the HCC group than in the non-HCC group of HBV-infected patients [17]. In addition, K130M+V131I HBx double mutation has been reported to increase in frequency with the liver disease progression in HBV-infected Taiwanese patients [18]. The functional role of HBx mutations in the development of liver complications was investigated as well. It was suggested that K130M+V131I HBx double mutation might contribute to HCC development by initiating the increased nuclear factor kappa B (NF-κB) activity [19].

As no data are available on HBx mutations contributing to the pathogenesis of liver disease in HBV-infected patients in the Saudi population, the goal of this study was to investigate the prevalence of HBx mutations associated with different progressive clinical phases of HBV, especially those that could be linked to the risk of development of cirrhosis and/or HCC in HBV-infected Saudi patients.

## RESULTS

### Basic characteristics of study subjects

In the present study, 424 HBV-infected Saudi patients were investigated. Table 1 shows the details of basic characteristics of the study participants. Age of the patients was an associated risk factor for HCC development (*p* < 0.0001). HCC patients had the highest average age followed by liver cirrhosis (LC) patients, whereas the inactive (IC) and active carrier (AC) groups showed similar values of average age. Different stages of HBV infection showed a predominance of male patients. Additional analyses were performed for female patients only and the results are shown in Supplementary Table 1. The serum ALT levels were more elevated in the AC, LC, and HCC patients than in the IC patients (*p* < 0.0001). The LC patients had highest ALT levels, while HCC and AC patients had similar ALT levels. Also, multiple comparison analyses were done for age and ALT as shown in Supplementary Table 2. The majority of the patients (79%) included in this study were infected with HBV genotype D (manuscript in preparation).

**Table 1:** Baseline characteristics of all subjects included in the study

Variables	Inactive (*n* = 245)	Active (*n* = 125)	Cirrhosis (*n* = 26)	HCC (*n* = 28)	*P* value^a^
Age (years)n^*^	39.543 ± 12.373	36.162 ± 11.203	51.938 ± 12.189	60.00 ± 9.356	< 0.0001
**Gender**^○^					
Male	165 (67.35%)	94 (75.2%)	20 (77%)	26 (93%)	0.022
Female	80 (32.65%)	31 (24.8%)	6 (23%)	2 (7%)	
BMI^*^	30.253 ± 18.537	27.469 ± 6.26	26.135 ± 4.802	24.512 ± 4.35	0.296
ALT^*^	36.689 ± 22.252	77.500 ± 77.286	104.688 ± 153.758	80.722 ± 76.439	< 0.0001

^*^Variables are expressed as Mean ± SD, ^○^ Variables are expressed as count (%).^a^One way Anova for continuous data, chi square and fisher exact test for categorical data.

### HBx mutations present in different stages of HBV infection

**Figure 1:**
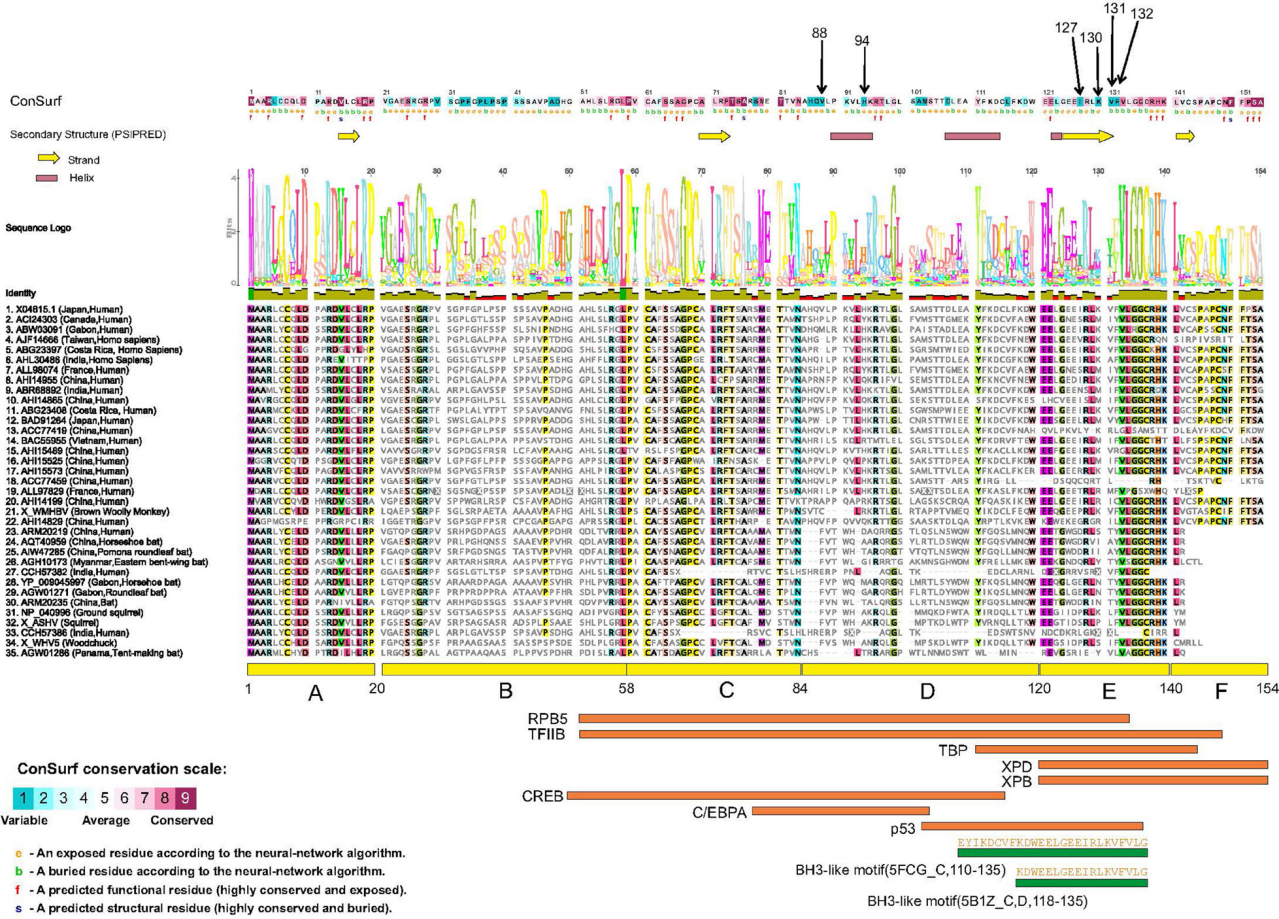
Structure-function analysis of the HBX protein Residue positions mutated in this work are indicated by arrows at the top. Consurf analysis results, with color coding ranging from most variable to most conserved residues are shown (See scale at bottom left). Below the ConSurf results, secondary structure predictions from PsiPred server are shown (helices and sheets). A multiple sequence alignment of 35 highly diverse HBX proteins from multiple species is shown. A sequence logo is placed right above the alignment. Columns in the alignment are colored according to > 75% conservation to the consensus sequence. Below the alignment, the six regions (A–E) defined by a previous analysis [6] are shown. Also shown are eight regions delineated for interaction with different transcription factors (RPB5, TFIIB, TBP, XPD, XPB, CREB, C/EBPA and p53). Shown at the bottom (green rectangles) are regions of HBX that have 3D structural information. The PDB codes and chain identifiers, and the sequences are shown.

The prevalence of all mutations in different clinical phases of HBV infection is shown in Supplemetary Figure 1 and summarized in Table 2. Most mutations with significantly different distribution between the groups are listed in Table 2. Three mutations showed an increasing trend from inactive HBV carriers to cirrhosis and HCC groups. These included mutations of isoleucine (I) to threonine (T) at position 127 (*p* < 0.0001), valine (V) to I at position 131 (*p* = 0.025), and phenylalanine (F) at position 132 to either tyrosine (Y), isoleucine (I), or arginine (R) (*p* < 0.0001). The prevalence of I127T mutation was 50% in HCC, 19.23% in LC, 13.6% in AC, and 11.84% in IC groups. V131I mutation was more prevalent in HCC (53.57%) than in LC (34.62%), AC (34.4%), and IC (26.94%). F132Y/I/R was present in 39.3% of HCC patients, while it showed low prevalence in LC (3.85%), AC (4%), and IC groups (4.49%). Another significant mutation at position 88 (substitution of V to F) was present in all the HCC samples, while only 65.38% of LC and 73.6% of AC and 73.06% of IC had this mutation (*p* = 0.011). Mutation of lysine (K) to methionine (M) at position 130 of HBx was observed in 50% of HCC patients. Histidine (H) at position 94 was substituted to tyrosine (Y) in 25% of HCC samples, while this mutation was present at low frequency in the other clinical groups (Table 2).

**Table 2:** Frequencies of HBx mutations in different progressive stages of HBV infection

Mutations	Inactive (*n* = 245)	Active (*n* = 125)	Cirrhosis (*n* = 26)	HCC (*n* = 28)	*P* value
S42P	191(77.96%)	95 (76%)	19 (73.07%)	27 (96.4%)	0.101
A47T	183 (74.7%)	95 (76%)	16 (61.54%)	26 (92.8%)	0.06
V88F	179 (73.06%)	92 (73.6%)	17 (65.38%)	28 (100%)	0.011
H94Y	22 (8.97%)	4 (3.2%)	3 (11.54%)	7 (25%)	0.003
I127T	29 (11.84%)	17 (13.6%)	5 (19.23%)	14 (50%)	< 0.0001
K130M	48 (19.59%)	37 (29.6%)	7 (26.92%)	14 (50%)	0.002
V131I	66 (26.94%)	43 (34.4%)	9 (34.62%)	15 (53.57%)	0.025
F132Y/I/R	11 (4.49%)	5 (4%)	1 (3.85%)	11 (39.3%)	< 0.0001

### HBx combinational mutations associated with clinical phases of HBV infection

We evaluated the combined effect of double and triple mutations in HBx protein on the development of different clinical stages of HBV infection (Table 3). One double mutation and two triple mutations were observed with increasing prevalence in IC, AC, LC, and HCC. A combinational mutation with substitutions of K to M at position 130 and V to I at position 131 was present in 16.33% of IC, 24% of AC, 26.93% of LC, and 46.42% of HCC patients. The association of this double mutation to HCC occurrence was investigated between non-HCC (IC+AC+LC) and HCC groups. The combinational mutation of K130M+V131I was significantly associated with the risk of HCC development (*p* = 0.001). Similarly, this double mutation showed a significant difference in distribution when the IC group was compared with the AC+LC+HCC group (*p* = 0.004) as well as when the AC group was compared with the HCC group (*p* = 0.017). A triple mutation with substitution of I to T at position 127, K to M at position 130, and V to Isoleucine (I) at position 131 of HBx was observed in 9.38% of IC, 8.8% of AC, 11.54% of LC, and 46.42% of HCC patients. This combinational mutation (I127T+K130M+V131I) was significantly associated with the risk of HCC development (*p* < 0.0001). I127T+K130M+V131I combinational mutation showed a significant difference in occurrence when the AC group was compared with the LC+HCC group (*p* < 0.0001) as well as when the HCC group was compared with the IC+AC+LC group (*p* < 0.0001). Another triple mutation with substitution of K to M, V to I, and F to Y at positions 130, 131, and 132, respectively, was present in 32.14% of HCC patients, while its frequency was very low in the IC group (0.41%). Both AC and LC groups did not have this combinational mutation (K130M+V131I+F132Y). K130M+V131I+F132Y combinational mutation was significantly associated with the risk of HCC development (*p* < 0.0001). This triple mutation also exhibited a significant association when the IC group was compared with the AC+LC+HCC group (*p* = 0.002).

**Table 3:** Prevalence of the combinational mutations in HBx present in different clinical stages of HBV infection

	DOUBLE MUTATION		TRIPLE MUTATIONS			
Groups	K130M + V131I	*p* value^#^	I127T+ K130M+ V131I.	*p* value^#^	K130M + V131I + F132 Y	*p* value^#^
**Inactive**	40 (16.33%)	0.004^*^	23 (9.38%)	0.072^*^	1 (0.41%)	0.002^*^
**Active**	30 (24%)	0.634^**^	11 (8.8%)	< 0.0001^**^	0 (%)	
**Cirrhosis**	7 (26.93%)	0.017^***^	3 (11.54%)	< 0.0001^***^	0 (0%)	
**HCC**	13 (46.42%)	0.001^****^	13 (46.42%)	< 0.0001^****^	9 (32.14%)	< 0.0001^****^

^*^Inactive compared to active, cirrhosis and HCC, ^**^Active compared Cirrhosis+ HCC groups, ^***^Active compared to HCC group. ^****^HCC compared to Inactive, active and cirrhosis.

^#^Since there are four categories in this analysis, the corrected significant *p* value is = 0.0125.

### Association between HBx mutations and biochemical factors

Univariate and multivariate logistic regression analyses were performed to assess the association between HBx mutations and progression of the disease. Univariate analysis showed that gender (*p* < 0.0001), HBV viral load (*p* < 0.0001), ALT (*p* = < 0.0001), mutation from K to M at amino acid (aa) 130 (*p* = 0.002) and V to I at aa131 (*p* = 0.042) had a significant positive correlation when the IC group was compared with AC+LC+HCC (Table 4). In addition, multivariate analysis showed a significant association with HBV viral load (*p* < 0.0001), ALT (*p* = 0.002) and the mutation from F to Y at aa132 (*p* = 0.007) (Table 4). Similarly, there were significant differences when the AC group was compared with LC+HCC patients. Univariate analysis showed that age (*p* < 0.0001), HBV viral load (*p* < 0.0001), mutation from A to S at aa47 (*p* = 0.047), mutation from H to Y at aa94 (*p* = 0.002), mutation from I to T at aa127 (*p* = 0.001), mutation from F to Y at aa132 (*p* = 0.001) and mutation from A to V at aa146 (*p* = 0.025) showed a significant correlation with HBV-related LC+HCC (Table 5). Furthermore, multivariate regression analysis revealed that HBV viral load (*p* < 0.0001), mutation from H to Y at aa94 with (*p* = 0.011), and mutation from F to Y at aa132 (*p* = 0.035) were independently associated with LC+HCC (Table 5). In addition, further analyses were performed for males and females separately (Supplementary Tables 3 and 4).

**Table 4:** Univariate and multivariate logistic regression analysis among inactive group vs active+cirrhosis+HCC groups

Univariate analysis					Multivariate analysis			
Variables	Odds ratio	95% C.I.		*P* value	Odds ratio	95% C.I.		*P* value
		Lower	Upper			Lower	Upper	
Age	1.008	0.993	1.022	0.293				
Gender	0.431	0.283	0.654	< 0.0001	0.683	0.355	1.316	0.255
Viral Load	1.473	1.338	1.622	< 0.0001	2.263	1.846	2.773	< 0.0001
ALT	1.035	1.024	1.046	< 0.0001	1.019	1.007	1.03	0.002
I127T	0.603	0.359	1.015	0.057	0.726	0.304	1.737	0.472
K130M	0.503	0.322	0.785	0.002	0.643	0.238	1.736	0.383
V131I	0.652	0.432	0.985	0.042	0.936	0.374	2.342	0.888
F132Y	0.403	0.158	1.032	0.058	0.118	0.025	0.553	0.007

**Table 5:** Univariate and multivariate logistic regression analysis among active group vs cirrhosis+ HCC groups

Univariate analysis					Multivariate analysis			
Variables	Odds ratio	95% C.I.		*P*-value	Odds ratio	95% C.I.		*P* value
		Lower	Upper			Lower	Upper	
Age	1.163	1.112	1.216	< 0.0001				
Gender	0.949	0.497	1.812	0.873				
ALT	0.999	0.996	1.003	0.69	1.001	0.996	1.006	0.654
Viral load	0.764	0.681	0.857	< 0.0001	0.490	0.371	0.647	< 0.0001
A47S	0.106	0.012	0.973	0.047	0	0	-	1
H94Y	0.155	0.046	0.517	0.002	0.084	0.013	0.561	0.011
I127T	0.267	0.125	0.568	0.001	0.333	0.108	1.020	0.054
F132Y	0.068	0.014	0.317	0.001	0.076	0.007	0.839	0.035
A146V	0.083	0.010	0.732	0.025	0.672	0.019	23.644	0.827

### Molecular/structural analysis of HBx

Two different methods were used to evaluate residue conservation (see methods) and variability in the HBX protein (described in methods). Both provided very similar results. All six positions that have been mutated in this work (88, 94, 127, 130, 131, 132) appear to lie in highly variable regions in the multiple sequence alignments (See Figure 1, color coded in dark blue in the ConSurf results panel at the top). None of these mutations changes any residue that is consistently conserved across all sequences, suggesting that the effect on protein structure itself is minimal. However, these may change (increase or decrease) binding to multiple different proteins. The effect of these mutations on interactions with any particular protein is unknown, as the same regions of HBX have been implicated in binding multiple targets (e.g. region E in the figure is known to interact with at least 6 targets, and four residues were mutated in this region).

Only a small region at the C-terminal of the HBX structure has been determined (PDB codes 5FCG [20], complexed with Bcl-2 and 5B1Z, complexed with Bcl-xL (unpublished) that suggest a BH3-like motif (also found in Bim, Bad proteins) in this region (Figure 1). Efforts to model the complete 3D structure of the HBX protein were not successful. However, the HHpred server suggested weak hits to the SCOP database (b.34.8.0 and b.34.8.1, both SH3-like barrel folds) in two regions (46–68 and 79–98). These hits however remain small, and unreliable.

### Sliding window analysis of amino acid Diversity in HBx

**Figure 2:**
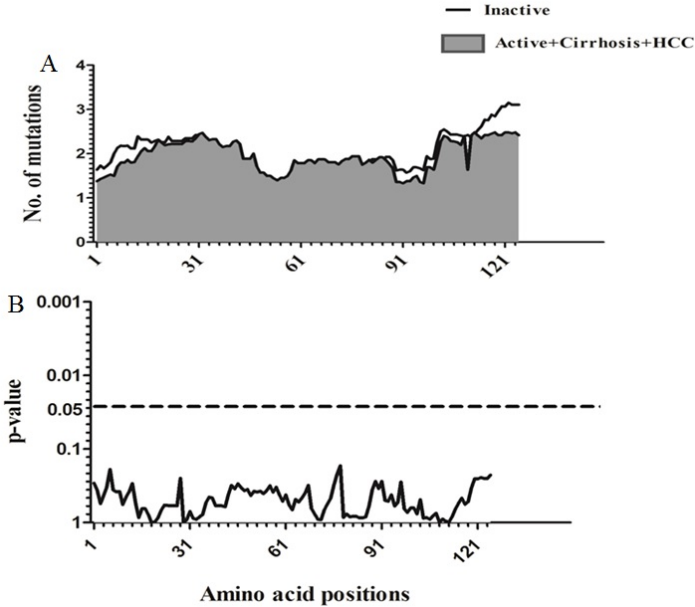
Comparison of amino acid variations in HBx using sliding window analysis Deduced amino acid sequences from IC patients were compared with sequences from AC+LC+HCC patients. The panel in (**A**) shows the mean number of amino acid mutations in IC patients (thick line) and AC+LC+ HCC patients (shaded area). Panel (**B**) shows the probability of observed differences in the amino acid mutations between IC and AC+ LC+ HCC which was calculated for each window by t test and was plotted as an inverted logarithmic scale. The dashed line indicates the *p* value of 0.05 for better visualization of the statistically significant values.

**Figure 3:**
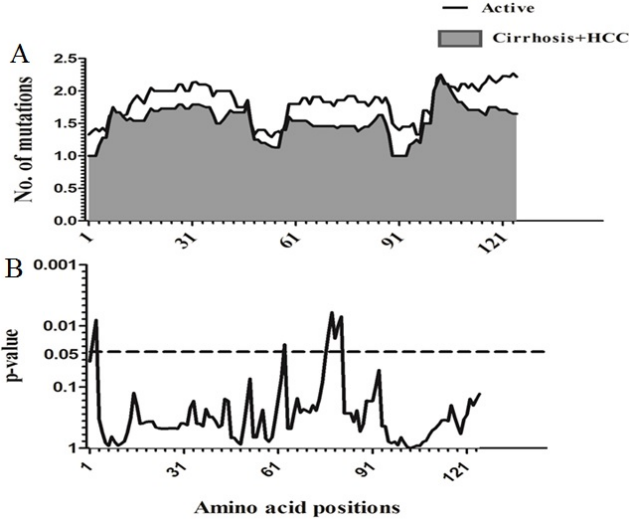
Comparison of amino acid variations in HBx using sliding window analysis Deduced amino acid sequences from AC patients were compared with sequences from LC+HCC patients. The panel in (**A**) shows the mean number of amino acid mutations in AC patients (thick line) and LC+ HCC patients (shaded area). Panel (**B**) shows the probability of observed differences in the amino acid mutations between AC and LC+ HCC which was calculated for each window by t test and was plotted as an inverted logarithmic scale. The dashed line indicates the *p* value of 0.05 for better visualization of the statistically significant values.

Sliding window analysis was used to assess the amino acid diversity between the different groups of patients in progressive stages of HBV infection. No significant variation was detected when the IC group was compared to AC+LC+HCC groups (Figure 2). However, when the AC group was compared with LC+HCC groups, the analysis revealed that the difference in the number of mutations was statistically significant in the regions extending from aa1 to aa3 (*p* = 0.008), at position aa63 (*p* = 0.02) and from aa76 to aa81 (*p* = 0.006) (Figure 3).

## DISCUSSION

HBV infection is one of the most important causes of human liver disease and continues to remain a medical concern in Saudi Arabia. There is a significant number of cases as the incidence rate of HBV infection was 14.7 per 100,000 Saudi inhabitants in 2013, with 23,236 cases recorded from 2009 to 2013 [21]. There is an emerging interest to explore various aspects of HBV infection and related liver complications within the Saudi population.

Many studies have shown that genetic variations in HBV genome influence clinical manifestations and progression of the HBV infection [22, 23]. HBx is a small regulatory protein with a plethora of activities influencing both viral and cellular genes. HBx is known to elicit an oncogenic potential by disrupting cell cycle regulation [7, 24]. HBx detected in HCC patients is known to frequently harbor mutations, which may play a role in the progression of HBV infection [12]. The present study identified specific HBx mutations and investigated the clinical correlation of these mutations and the progression of HBV infection. Eight positional single mutations within the HBx protein playing a role in advancement of liver disease were identified in this study. Two mutations (S42P and A47T) present within the B-cell epitope region did not show any significant association with the clinical stages of HBV infection. This was inconsistent with a previous Indian study reporting a significant association of mutation at codon 47 of HBx with increased disease severity [15]. Similarly, mutation at position 88 of HBx protein was associated with liver cirrhosis in Indian population [15], while a Chinese study has found a mutation at amino acid 88 more frequent in HCC samples [16]. Our study found mutation V88F to be highly prevalent (100%) in the HCC group but its prevalence in LC was less than in both IC and AC groups in the Saudi population. As suggested by Malik et al. [14], the difference in association of mutations with severe symptoms of HBV infection varies across different ethnic populations. Several previous studies on HBV genotypes have reported that H94Y, I127T, K130M, V131I, and F132Y/I/R mutations are frequently considered as associated risk factors of HCC and may promote progression of liver impairment [14, 17, 25, 26]. The results in our study also showed a significant association of these mutations with severity of liver disease. The prevalence of I127T, V131I, and F132Y/I/R mutations showed an increasing trend with increase in severity of disease from the IC to the HCC group. Malik et al. [14] have found an increased prevalence of V131I and K130M mutations proportional to progressive forms of HBV infection in patients. Further, it was observed that H94Y and F132Y/I/R mutations were present with high frequency only in the HCC group, suggesting that these mutations were associated with an increased risk of HCC. Shinkai et al. [17] also have found H94Y mutation to be associated with HCC in patients with HBV genotype C2. However, some studies in the Indian population have reported that H94Y mutation was present at high frequencies in both LC and HCC groups, suggesting that population may increase or decrease the effect of this mutation on liver disease [25, 27]. In addition, analysis of I127T mutation showed that its frequency was higher in the HCC (50%) than in the LC (19.23%) group. These results were consistent with a previous study that has found a higher percentage of this mutation in the HCC (53%) than in the LC (19%) group [28]. A study in Mongolian patients with HBV genotype D has shown that I127T mutation is significantly linked to HCC development [29]. The biological mechanisms elucidating the association of V88F, H94Y, I127T, K130M, V131I, and F132Y/I/R mutations with the pathogenesis of HBV infection are unclear. These mutations, located in regions D (∼85-119 aa) and E (∼120-140 aa) functional domains of HBx, are associated with nuclear transactivation and signal transduction [6]. Therefore, these mutations may be responsible for modulating the transactivation property of HBx that is known to be associated with hepatocarcinogenesis [30, 31].

The present study also investigated the association of combinational mutations in the HBx protein with the progression of HBV infection. The combinational mutations analyzed could act synergistically to increase severity of liver disease. K130M+V131I double mutation showed a statistically significant increase in prevalence from IC to HCC stage. This double mutation was present at high frequency in the HCC (46.42%) and in the LC group (26.93%), indicating a probable role in the advancement of liver disease. These results were in agreement with previous studies reporting an association between K130M+V131I mutation and disease progression [19, 25, 26]. K130M+V131I double mutation is a common mutation present across different populations. For instance, it has shown an increase in frequency with the advancing clinical phases in Taiwanese [18], Chinese [28], and Indian populations [14]. However, the prevalence of this mutation in HCC varied across the populations (85%, 64%, and 45% in Taiwanese, Chinese, and Indians, respectively). The prevalence of this double mutation in the Saudi HCC patients (46.42%) analyzed in this study was similar to the percentage in Indian patients (45%) [14], which may be due to both having a predominance of HBV genotype D. The mechanism by which K130M+V131I double mutation leads to progression of HBV related liver disease is not fully understood. This double mutation is present within the C-terminal of HBx protein, and several studies related mutations in C-terminal to cell proliferation and transformation contributing to hepatocarcinogenesis [31]. Lee et al. have suggested that K130M+V131I double mutation increases NF-κB activity, which is known to play an important role in liver function and in regulation of oncogenic genes, leading to carcinogenesis [19]. Another study has proposed that K130M+V131I double mutation causes rapid progression to cirrhosis in HBV patients and, thus, has an indirect role in the development of HCC [32].

I127T+K130M+V131I triple mutation was significantly associated with AC, LC, and HCC stages. I127T+K130M+V131I triple mutation was present in increasing proportion with advancing clinical phases of HBV infection from AC to HCC. However, this mutation was observed at high frequency only in HCC patients (46.42%). A recent study on HBV genotype D has found this triple mutation to be associated with HCC patients (67%) [25]. Previous data have established the specific presence of mutation at position 127 along with the double mutation at positions 130 and 131 [33], a strong association between K130M+V131I mutation and the presence of a polar mutation at position 127 (I to T) [33]. Takahashi et al. [34] have demonstrated that the mutation at position 127 arises later than the K130M+V131I double mutation in the course of HBV infection. As also stated by Iavarone et al. [33], this suggested that the presence of K130M+V131I double mutation favors the occurrence of a polar mutation at position 127. Another combinational mutation observed in our study was K130M+V131I+F132Y. This triple mutation was present at very low frequency in IC (0.41%) and increased to 32.14% in HCC patients, while was completely absent in AC and LC stages. This mutation was significantly associated with the development of HCC, though its prevalence in HCC was less than that of K130M+V131I (46.42%) and I127T+K130M+V131I (46.42%) mutations. These results were consistent with an earlier study that has found K130M+V131I double mutation with a mutation at position 132 to be associated with development of HCC [35]. Interestingly, in both cases where K130M+V131I double mutation was associated with adjacent mutation at position 127 or 132, the frequency of the mutation showed a striking increment from non-HCC phase to HCC phase, whereas that of only K130M+V131I double mutation was already high in non-HCC patients. This suggested that the existence of triple mutations might burden the life cycle of HBV and accelerate the development of HCC. Similar observations were made by Lee et al. [19] who have stated that triple mutations could disrupt protein tertiary structure of HBx.

Multivariate logistic regression analysis was informative as it confirmed our finding that H94Y, I127T and F132Y mutations in HBx can be useful as independent prognostic markers of HBV-associated liver disease evolution and advancement, especially when other contributing factors, such as viral load, exist. Such finding could yield better management of HBV-infected patients before progression to liver complications. However, these findings should be interpreted with caution as the number of subjects who showed LC and HCC is limited in this study.

In conclusion, the present study confirmed that mutations in HBx are frequent and found in patients exhibiting different manifestation of HBV-associated liver complications. By comparing the full-length HBx sequences, several point and combinational mutations correlating with severity and progressive clinical phases of HBV infection were identified. Although the significant identified single mutations correlated well with the development of HCC, the synergistic effect of combinational mutations may have a role in accelerating progression to HCC. These mutations can be useful in predicting the clinical outcome of HBV-infected patients and may serve as early markers of high risk of developing HCC. Prospective studies exploring the functional mechanism of the mutations identified in this study can improve the understanding of the progression of liver disease in HBV-infected patients in the Saudi population.

## MATERIALS AND METHODS

### Subjects

A total of 424 Saudi patients infected with HBV were analyzed in this study. The patients were recruited from three hospitals in Riyadh, Saudi Arabia, including King Faisal Specialist Hospital and Research Center (KFSHRC), King Khalid University Hospital (KKUH), and Prince Sultan Military Medical City (PSMMC). The study protocol was approved by the institutional review boards of all centers and conformed to the 1975 Declaration of Helsinki. All subjects that participated in this study signed an informed consent prior to the enrollment in the study.

Chronic HBV infection was diagnosed by the repeated detection of HBsAg over a period of 6 months. Patients were grouped into four different categories based on disease severity as follows: Case I - Inactive HBV carriers (IC), including patients that were positive for HBsAg and negative for HBeAg, with persistently normal serum alanine aminotransferase (ALT) levels; Case II - Active HBV carriers (AC), including patients that were positive for HBsAg, with elevated serum ALT levels with no evidence of any liver complication; Case III - patients with HBV infection and liver cirrhosis (LC) confirmed by liver biopsy, clinical, biochemical, or radiological evidence of cirrhosis; Case IV - patients with HCC diagnosed by computed tomography and/or magnetic resonance imaging of the liver, according to the published guidelines for the diagnosis and management of HCC [36].

### Extraction of nucleic acids from serum

Viral DNA was extracted from 200 μL of each sample using the QIAamp^®^ MinElute™ Virus Spin Kit (Qiagen, Velencia, CA, USA) according to the manufacturer's protocol.

### HBx gene amplification

The sequence for *HBx gene* (accession number X04615 [37]) was used as the reference sequence for the analysis described in this work. A nested PCR protocol was used to amplify and detect mutations using primers covering the entire region of the X gene (Supplementary Table 5). Nested primers were tagged with M13 sequence for direct sequencing after amplifications. GoTaq^®^ Green Master Mix was used (Promega, Madison, WI, USA). First-round PCR was performed with 5 μL of extracted DNA, 12.5 picomoles of each primer (HBxF1 and HBxR1), and Green Master Mix up to a total volume of 25 μL. The PCR condition consisted of an initial denaturation step of 3 min at 94°C, followed by 35 cycles of amplification (94°C for 30 sec, 55°C for 30 sec, and 72°C for 1 min), and final extension at 72°C for 7 min. In the second round of PCR, 2 μL of the first-round product was re-amplified using the same reaction mixture composition containing HBxF2 and HBxR2 primers. The PCR conditions used were the same as in the first round with only the number of cycles reduced to 30. A negative control was included in the amplification process. After amplification, 10 μL of the PCR product were analyzed on a 2% agarose gel stained with ethidium bromide, and visualized under UV light. The PCR product of the expected size was excised from the gel and purified using QIAquick Gel Extraction kit (Qiagen, Velencia, CA, USA).

### DNA sequencing

Purified PCR products were directly sequenced with the tagged M13 sequence forward and reverse primers in both directions. The sequencing was performed on the ABI 3730 Genetic Analyzer using BigDye^®^ Terminator v3.1 Cycle Sequencing Kit (Applied Biosystems, Foster City, CA, USA). Sequences of the whole HBx gene (465 nt; 154 aa) were assembled and edited using Lasergene software package (DNASTAR, Madison, WI, USA), and aligned with the ClustalX algorithm included in the Megalign module (DNASTAR).

### Molecular analysis and protein structure prediction

The HBx protein sequence was submitted to the ConSurf server [38] (http://consurf.tau.ac.il/2016/). As no 3D structure is available for this protein, the analysis was run in ConSeq mode. HMMER [39] was used to retrieve homologs from the UNIREF90 database (evalue 1e^−3^, minimum and maximum % identity between homologs 35% and 95% respectively). A total of 147 sequences were retrieved and a multiple sequence alignment was built using MAFFT [40].

The multiple sequence alignment was constructed by retrieving homologous protein sequences using BLASTP against the NCBI NR database (evalue 1e^−3^, matrix BLOSUM45). Approximately 5900 sequences were identified as significant hits. These were aligned using MAFFT [40]. Sequences with N- or C-terminal overhangs were removed and a final set of 5853 sequences were realigned. The alignment was filtered for the most diverse sequences (using the program hhfilter available from the hhsearch package [41]. Secondary structure prediction was performed using the PsiPred server [42].

To predict the three dimensional (3D) structure of the HBx protein, the amino acid sequence was submitted to the Phyre2 server (www.sbg.bio.ic.ac.uk/∼phyre2/) and the HHpred server (https://toolkit.tuebingen.mpg.de/) in an attempt to identify distant homologs and create a model. While the Phyre2 server returned a putative model, the model quality was low (only 16% residues modelled at high confidence) and thus unreliable.

### Statistical analyses

The Statistical Package for the Social Sciences (SPSS) version 20.0 (SPSS Inc., Chicago, IL, USA) was used for statistical analyses. Age, ALT, and body mass index (BMI) were expressed as mean ± standard deviation. The Chi square test and Fisher's exact test were used to compare categorical data. For all tests, *p* values ≤ 0.05 were considered significant. Only variables that reach statistically significant values (i.e. *p* ≤ 0.05) in univariate analysis were analyzed in multiple logistic regression analysis. DnaSP (version 5.10.1) [43] and prism software GraphPad Prism (version 7.0), GraphPad Software, La Jolla California USA) were used to generate a sliding windows analysis.

## SUPPLEMENTARY MATERIALS FIGURE AND TABLES


