# Supplementary material for: Hepatitis B virus (HBV) X gene mutations and their association with liver disease progression in HBV-infected patients
Source: Oncotarget. 2017 Nov 6;8(62):105115–25. doi: 10.18632/oncotarget.22428 (PMC5739625; doi:10.18632/oncotarget.22428)
Supplement: Supplementary file 1 [file oncotarget-08-105115-s001.pdf]

Hepatitis B virus (HBV) X gene mutations and their association with liver disease progression in HBV-infected patients

SUPPLEMENTARY MATERIALS

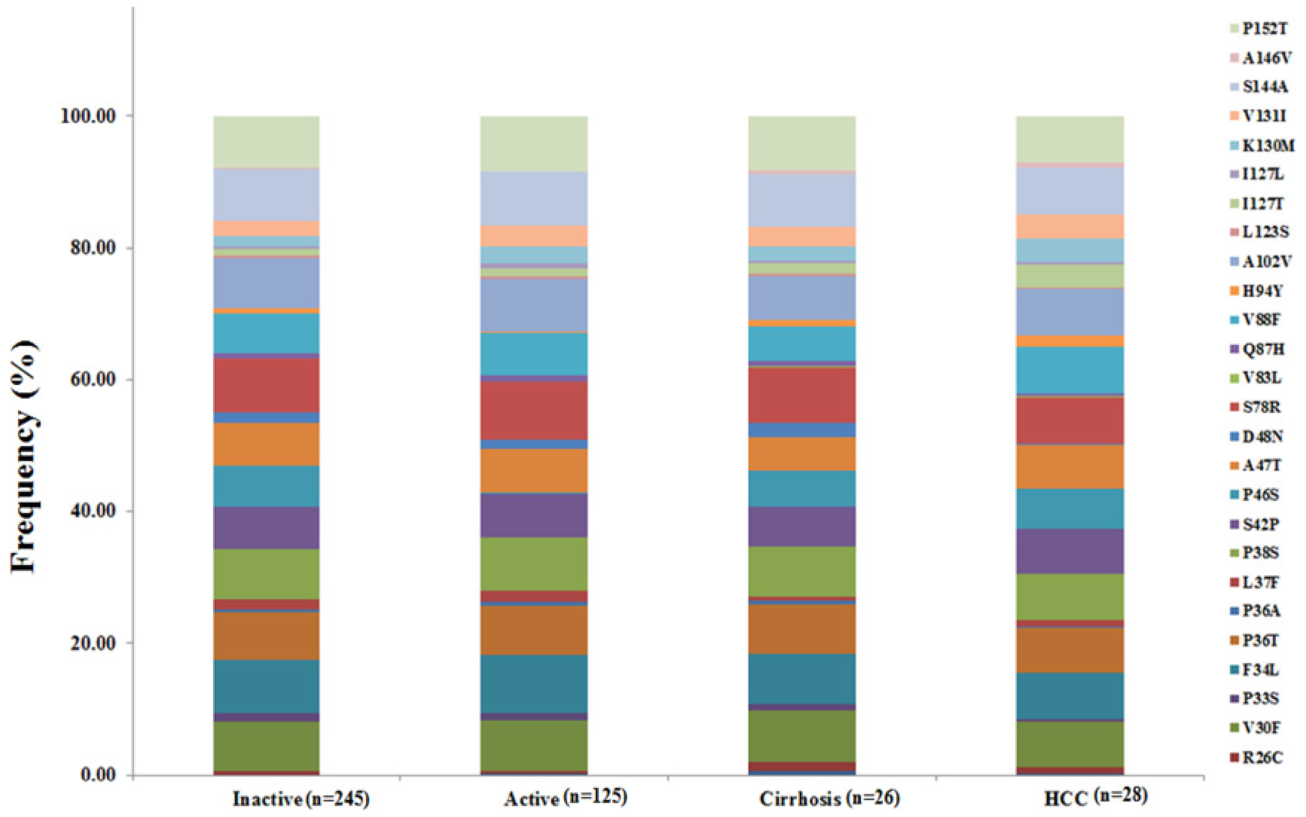

Supplementary Figure 1: All HBx mutations classified according to clinical stages of HBV infection.

Supplementary Table 1: Baseline characteristics of subjects included in the study based on female gender

Variables	Inactive (*n* = 80)	Active (*n* = 31)	Cirrhosis+HCC (*n* = 8)	*P* value[a]
**Age (years)[*]**	40.16 ±12.15	33.42 ± 8.98	54.33 ± 20.59	0.0004
**BMI[*]**	31.84 ± 24.22	28.06 ± 6.49	28.42 ± 3.2	0.766
**ALT[*]**	27.84 ± 13.46	54.26 ± 53.30	62.67 ± 14.64	< 0.0001
**HBV viral load(log10)◊**	2.52 (1.45=3.36)	6.03 (4.57=7.81)	1.76 (0.88–2.73)	< 0.0001

[*]Variables are expressed as Mean ± SD, [◊]Variables are expressed as median interquartile range (25th-75th). [a]One way Anova and nonparametric test for continuous data.

Supplementary Table 2: Multiple comparison analysis of age and ALT in HBV-infected groups

Tukey test<br>Dependent Variable	HBV categories		Std. Error	*P* value	95% Confidence Interval<br>Lower Bound	Upper Bound
AGE	1	2	1.320	.001	1.4909	8.3035
		3	2.351	.000	–17.2792	–5.1488
		4	2.427	.000	–26.3025	–13.7781
	2	1	1.320	.001	–8.3035	–1.4909
		3	2.464	.000	–22.4671	–9.7552
		4	2.537	.000	–31.4817	–18.3932
	3	1	2.351	.000	5.1488	17.2792
		2	2.464	.000	9.7552	22.4671
		4	3.197	.031	–17.0731	–.5795
	4	1	2.427	.000	13.7781	26.3025
		2	2.537	.000	18.3932	31.4817
		3	3.197	.031	.5795	17.0731
ALT	1	2	8.403	.000	–76.0422	–32.6612
		3	15.108	.004	–90.6557	–12.6567
		4	16.409	.063	–83.2046	1.5088
	2	1	8.403	.000	32.6612	76.0422
		3	15.882	.998	–38.3018	43.6928
		4	17.124	.860	–30.6991	57.7068
	3	1	15.108	.004	12.6567	90.6557
		2	15.882	.998	–43.6928	38.3018
		4	21.234	.957	–44.0040	65.6206
	4	1	16.409	.063	–1.5088	83.2046
		2	17.124	.860	–57.7068	30.6991
		3	21.234	.957	–65.6206	44.0040

Supplementary Table 3: Univariate and multivariate logistic regression analysis among inactive group vs active+cirrhosis+ HCC groups in male gender

	Univariate analysis				Multivariate analysis			
Variables	Odds ratio	95% C.I.		*P*-value	Odds ratio	95% C.I.		*P* value
		Lower	Upper			Lower	Upper	
Age	1.011	0.995	1.028	0.183				
BMI	0.954	0.91	0.999	0.046	0.937	0.881	0.998	0.042
ALT	1.028	1.017	1.039	< 0.0001	1.011	1.001	1.021	0.026
Viral load	1.404	1.264	1.561	< 0.0001	1.928	1.564	2.375	< 0.0001
A47S	1.201	0.316	4.56	0.788				
A47T	0.651	0.377	1.123	0.123				
V88F	0.684	0.408	1.147	0.15				
H94Y	1.271	0.595	2.715	0.536				
A102V	1.298	0.525	3.208	0.572				
I127T	0.649	0.36	1.172	0.152				
K130M	0.536	0.318	0.903	0.019	0.43	0.191	0.969	0.042
V131I	0.753	0.466	1.215	0.245				
S144A	0.827	0.321	2.131	0.694				
P152T	0.571	0.187	1.744	0.325				

Supplementary Table 4: Univariate and multivariate logistic regression analysis among inactive group vs active+cirrhosis+ HCC groups in female gender

	Univariate analysis				Multivariate analysis			
Variables	Odds ratio	95% C.I.		*P*-value	Odds ratio	95% C.I.		*P* value
		Lower	Upper			Lower	Upper	
Age	0.986	0.955	1.017	0.363				
BMI	0.978	0.912	1.048	0.524				
ALT	1.062	1.031	1.093	< 0.0001	1.081	1.023	1.143	0.006
Viral load	1.629	1.311	2.025	< 0.0001	4.493	2.036	9.918	< 0.0001
A47S	2.079	0.127	34.143	0.608				
A47T	2.662	1.17	6.056	0.02	1.378	0.004	433.65	0.913
V88F	2.662	1.17	6.056	0.02	0.393	0.001	120.07	0.749
H94Y	2.533	0.286	22.46	0.404				
A102V	0.486	0.098	2.408	0.377				
I127T	0.611	0.196	1.903	0.395				
K130M	0.462	0.193	1.103	0.082	1.425	0.199	10.221	0.724
V131I	0.533	0.235	1.211	0.133				
S144A	1.387	0.222	8.663	0.726				
P152T	0.395	0.045	3.5	0.404				

Supplementary Table 5: Primers used to amplify the HBx region

Primers	Position	Sequence	size
**Round 1**			
HBxF1	973–941	5′ ATT GAT TGG AAA GTM TGT M 3′	969 bp
HBxR1	1919–1941	5′ TCC ACA GTA GCT CCA AAT TCT TT 3′	
**Round 2**			
HBxF2	1287–1305	5′ CGC TTG TTT TGC TCG CAG C 3′	597 bp
HBxR2	1865–1883	5′ GGC ACA GCT TGG AGG CTT G 3′	
